# Laxative use and 28-day mortality in critically ill sepsis patients: a retrospective cohort study using MIMIC-IV (v3.1)

**DOI:** 10.1186/s40560-025-00797-9

**Published:** 2025-05-21

**Authors:** Yan Liang, Feiyi Xu, Hao Zhang, Jiang Li, Wei Chen, Qilin Yang, Cheng Lin, Xiaomin Dong

**Affiliations:** 1 The First Affiliated Hospital Of Guilin Medical University, No. 15, Lequn Road, Xiufeng District, Guilin, Guangxi Province China; 2 Guilin Medical University, No. 1 Zhiyuan Road, Lingui District, Guilin, Guangxi China; 3 Guilin People’s Hospital, No. 12, Wenming Road, Xiufeng District, Guilin City, Guangxi Province China; 4 The Second Affiliated Hospital of Guangzhou Medical University, No. 250 Changgang East Road, Haizhu District, Guangzhou, China

**Keywords:** Sepsis, 28-day mortality, Laxatives, Docusate Sodium, MIMIC-IV (version 3.1) database, Critical care

## Abstract

**Objective:**

This study investigates the impact of four laxatives-Senna, Docusate Sodium, Polyethylene Glycol, and Lactulose on 28-day mortality, ICU-free days, ventilator-free days, bowel recovery, and Clostridium difficile (C-diff) infection in critically ill sepsis patients to identify optimal bowel management strategies for improving survival and recovery.

**Methods:**

Using the MIMIC-IV database (v3.1), we analyzed 7163 ICU sepsis patients (median age: 67.5 years; 63% male), assessing 28-day mortality, ICU-free days, vasopressor-free days, ventilator-free days, bowel sound recovery, and C-diff incidence, with propensity score matching and multivariable adjustments for confounders, alongside subgroup analyses by sex, age, Charlson Comorbidity Index, and Sequential Organ Failure Assessment score.

**Results:**

Docusate Sodium was associated with significantly lower 28-day mortality (adjusted HR: 0.43; 95% CI 0.36–0.52), more ICU-free days, and better bowel recovery compared to Senna, while Lactulose was linked to higher mortality (adjusted HR: 1.82; 95% CI 1.45–2.27), fewer ICU-free days, and increased C. difficile risk, with subgroup analyses confirming these trends across sex, age, and comorbidity strata.

**Conclusion:**

Docusate sodium appears to be a safer and more effective bowel management option for critically ill patients with sepsis. In contrast, the association between lactulose use and adverse outcomes may primarily reflect the severity of underlying liver disease rather than a direct drug effect. These findings underscore the importance of individualized laxative selection based on patients’ clinical context in critical care practice.

**Supplementary Information:**

The online version contains supplementary material available at 10.1186/s40560-025-00797-9.

## Introduction

Sepsis continues to be a formidable global health crisis, accounting for over 11 million deaths annually from approximately 49 million cases, and remains one of the leading causes of mortality in intensive care units (ICUs) worldwide [1]. Despite advances in critical care, mortality rates remain alarmingly high, often surpassing 30% in ICUs across various regions, including China [2]. Sepsis patients frequently face significant long-term complications, a recent meta-analysis showed that 41.9% of sepsis deaths had intestinal dysfunction [3]. These statistics emphasize the persistent challenges in sepsis management and highlight the pressing need for innovative strategies that can reduce mortality and enhance patient recovery.

Within ICU settings, laxatives are routinely used to address constipation and preserve gut function. These agents work by altering the physical or chemical properties of intestinal contents or stimulating motility directly [4]. Commonly employed laxatives, including osmotic agents (e.g., Lactulose), stimulants (e.g., Senna), and surfactants (e.g., Docusate Sodium), offer potential benefits such as improved bowel function and reduced complications like feeding intolerance [5]. However, their use in septic patients has sparked concern, with emerging evidence pointing to possible risks, such as increased susceptibility to life-threatening Clostridioides difficile (C-diff) infections [6, 7].

Despite these concerns, the relationship between laxative use and sepsis outcomes has received relatively limited scrutiny [8]. Preliminary findings suggest that while some laxatives, like Docusate Sodium, may mitigate systemic inflammation and improve survival, others, such as Lactulose, could exacerbate mortality risks due to side effects like electrolyte imbalances and gut irritation [9]. Yet, these insights remain inconclusive, hindered by limited, agent-specific studies.

This study harnesses the expansive MIMIC-IV (v3.1) database to comprehensively evaluate the effects of various laxatives on sepsis outcomes, including 28-day mortality, ICU-free days, and bowel sound recovery. By employing advanced methodologies such as propensity score matching and multivariable adjustments, we aim to provide robust evidence to guide clinical decision-making, refine bowel management strategies, and improve prognosis for sepsis patients in ICUs.

## Methods

### Data source

Data were obtained from the MIMIC-IV (version 3.1) database [10, 11]. This is a publicly available, de-identified dataset containing comprehensive health records of patients admitted to the ICUs at the Beth Israel Deaconess Medical Center between 2008 and 2022. MIMIC-IV includes detailed patient demographics, vital signs, laboratory results, comorbidities, treatments, and clinical outcomes, allowing for robust analyses of critically ill populations. One of the authors (XMD) accomplished the National Institutes of Health’s web-based course “Protecting Human Research Participants” (Record ID: 57459186) and was approved to access the database to extract data. To ensure patient privacy, all data were de-identified, and consequently, the requirement for informed consent was waived by the ethical committee of Beth Israel Deaconess Medical Center. This study adheres to the guidelines of the Strengthening the Reporting of Observational Studies in Epidemiology (STROBE) statement [12] and complies with the principles outlined in the Declaration of Helsinki.

### Patient inclusion

**Fig. 1:**
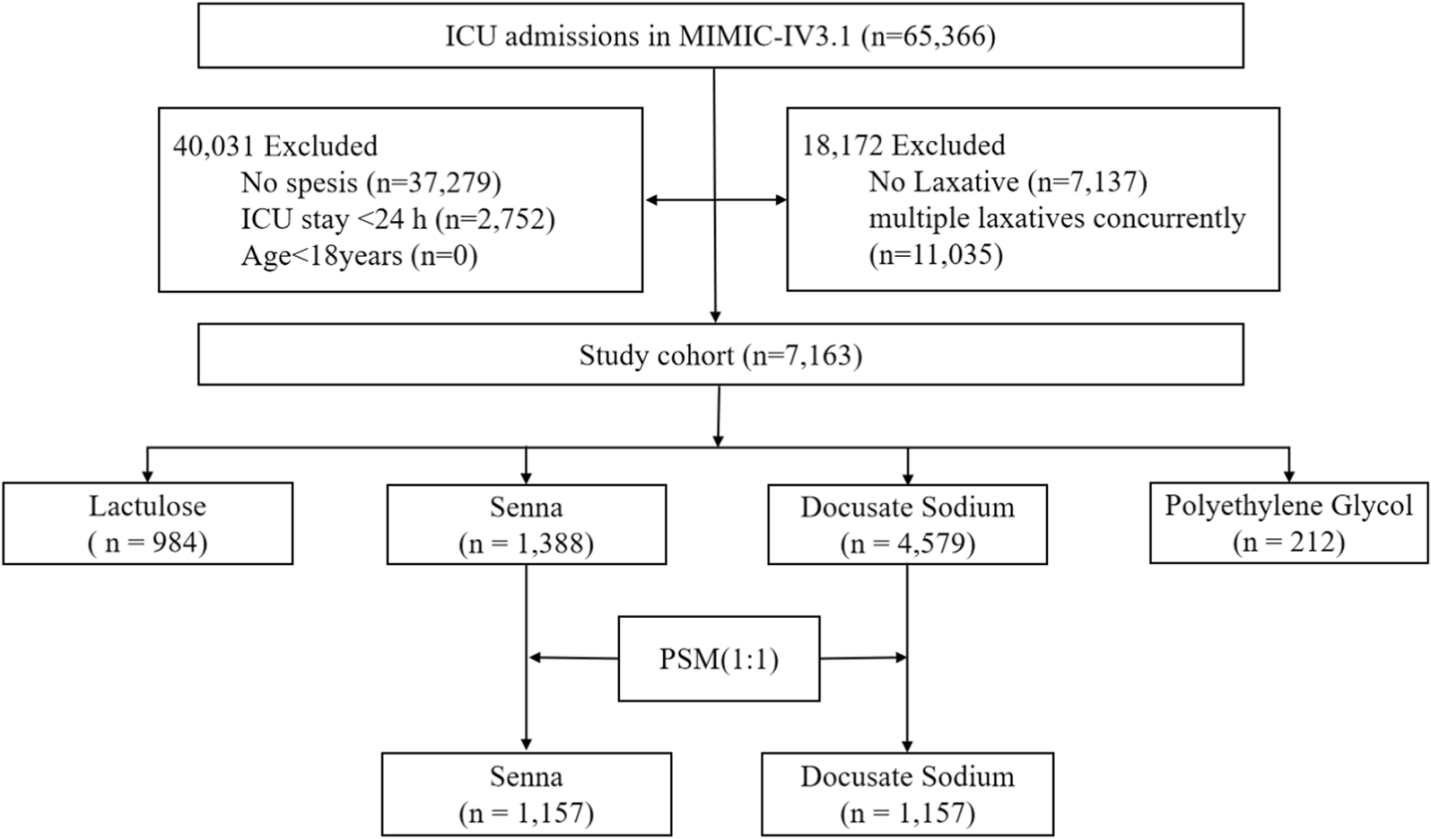
Flowchart of study patients

ICU patients diagnosed with sepsis were included in this study. Sepsis was defined according to Sepsis-3 criteria, which include suspected infection and organ dysfunction as indicated by a Sequential Organ Failure Assessment (SOFA) score ≥ 2. The exclusion criteria were as follows: (1) those who were not admitted to the ICU for the first time; (2) ICU stay ≤ 24 h; (3) received multiple laxatives concurrently (included Senna, Docusate Sodium, Polyethylene Glycol [PEG], or Lactulose); (4) Did not receive any of the four study laxatives during their ICU stay (Fig. 1).

### Variable extraction

Key variables were extracted from the MIMIC-IV database, including patient demographics (age, sex, and race), vital signs (heart rate, mean blood pressure, respiratory rate, temperature, and SpO_2_), laboratory values (e.g., glucose levels), comorbidities (e.g., hypertension, liver disease, myocardial infarction) and disease Severity Scores [Charlson Comorbidity Index (CCI), Acute Physiology Score III (APS III), Simplified Acute Physiology Score II (SAPS II), Oxford Acute Severity of Illness Score (OASIS), and SOFA].

All laboratory variables and disease severity scores were obtained from data recorded at the first instance after the patient’s admission to the hospital. Covariates with missing values exceeding 20% were excluded. Covariates with less than 20% missing data were processed using the multiple imputation scheme of the Free Statistics software version 2.0 and the statistical software package R 4.3.2.

### Outcomes

The primary outcome was 28-day mortality, defined as all-cause mortality within 28 days of ICU admission. Secondary outcomes included ICU-free days, vasopressor-free days, ventilator-free days within 28 days, and bowel sound recovery, which was defined as the transition from weak or absent bowel sounds at ICU admission to the presence of normal or hyperactive bowel sounds at ICU discharge. C-diff incidence was also evaluated as a safety outcome. All outcomes were compared across the four laxative groups: Senna, Docusate Sodium, Polyethylene Glycol, and Lactulose.

### Statistical analysis

Patient baseline characteristics were summarized using medians and interquartile ranges (IQRs) for continuous variables and frequencies and percentages for categorical variables. Group differences were assessed using the Kruskal–Wallis test for continuous variables and the chi-square test for categorical variables.

Kaplan–Meier survival curves were constructed to show survival probabilities across laxative groups, and log-rank tests were conducted to compare survival distributions between groups. Multivariate Cox analyses assessed 28-day mortality. Multivariate Cox proportional hazards regression models included clinically relevant variables. The final model variables were carefully selected based on the number of events available.

Three progressively adjusted models were developed to assess the association between laxative use and clinical outcomes, including 28-day mortality and secondary outcomes: Model 1: Adjusted for baseline demographics, including age, sex, and race. Model 2: Included additional adjustments for key physiological indicators, such as heart rate, mean arterial pressure (MAP), respiratory rate, temperature, SpO_2_, and glucose levels. Model 3: Further adjusted for comorbidities, including myocardial infarction, dementia, cerebrovascular disease, chronic pulmonary disease, peptic ulcer disease, liver disease, diabetes, paraplegia, renal disease, obesity, malignant cancer, hypertension, and congestive heart failure.

Before conducting the primary analysis comparing different laxative agents, we performed a preliminary comparison between patients who received any laxative treatment (n = 7163) and those who did not (n = 7137). Multivariable Cox proportional hazards models were used to assess 28-day mortality. The detailed results are provided in Supplementary Tables 1–4.

Secondary outcomes were analyzed using the following models: Bowel Sound Recovery: Due to the low event rate of C-diff infection, a parsimonious model (Model 1), adjusting only for age, sex, and race, was used to avoid overfitting and unstable estimation. In contrast, for other outcomes with sufficient events, fully adjusted models (Model 3) were applied. Analyzed using Model 3 to account for comprehensive confounders. C-diff Infection: Analyzed using Model 1, which adjusted for baseline demographics. ICU-Free Days, Vasopressor-Free Days, and ventilator-free days: Analyzed using Model 3 to provide robust adjustment for confounders. Binary outcomes (e.g., bowel sound recovery, C-diff infection) were analyzed using multivariable logistic regression models to estimate odds ratios (ORs) with 95% confidence intervals (CIs). Continuous outcomes (e.g., ICU-free days, vasopressor-free days, ventilator-free days) were analyzed using linear regression models to calculate adjusted mean differences and corresponding 95% CIs.

To minimize confounding, propensity score methods were implemented. Given the unbalanced sample sizes of multiple groups and to minimize sample attrition, we selected only the two groups with the largest sample sizes for PSM. Three-group PSM comparisons were not performed due to significant baseline imbalances, particularly the disproportionately high prevalence of liver disease in the lactulose group. Even with iterative matching approaches, achieving adequate covariate balance between groups would remain unfeasible. A 1:1 propensity score matching (PSM) algorithm was applied to compare patients receiving Docusate Sodium versus Senna, ensuring balance in baseline characteristics. Adequate balance was confirmed using a standardized mean difference (SMD) threshold of < 0.1.

The primary outcome, 28-day mortality, was further examined in predefined subgroups to evaluate potential effect modification by key patient characteristics. Subgroups were stratified by sex (male vs. female), age (< 65 vs. ≥ 65 years), Charlson Comorbidity Index (CCI < 6 vs. ≥ 6) [13], and SOFA score (< 6 vs. ≥ 6) [14]. The significance of interaction effects was determined using P values for interaction terms.

All statistical analyses were performed using R software (version 4.2.3; The R Foundation, http://www.R-project.org) and Free Statistics software version 2.0. A two-tailed P-value of less than 0.05 was considered statistically significant.

## Results

### Baseline characteristics

This study included 7163 ICU patients with sepsis, categorized into four groups based on laxative use: 1388 received Senna, 4579 received Docusate Sodium, 212 received PEG, and 984 received Lactulose (Table 1). The median age of the cohort was 67.5 years (IQR: 57.9–77.0). Patients in the Lactulose group were the youngest (median 58.5 years, IQR: 50.6–66.0), while those in the Polyethylene Glycol group were the oldest (median 72.6 years, IQR: 61.7–81.5). Males comprised 63.02% of the total cohort, with the highest proportion in the Docusate Sodium group (66.52%) and the lowest in the Senna group (53.17%).

**Table 1:** Baseline Characteristics of Patients by Treatment Group

Characteristics	Senna (reference, n = 1388)	Docusate Sodium, (n = 4579)	Polyethylene Glycol, (n = 212)	Lactulose (n = 984)	P
Age, median (IQR)	71.1 (58.8–82.4)	68.5 (60.0–77.0)	72.60 (61.7–81.5)	58.5 (50.6–66.0)	< 0.001
Race white, n (%)	877 (63.18)	3378 (73.77)	136 (64.15)	632 (64.23)	< 0.001
Male, n (%)	738 (53.17)	3046 (66.52)	123 (58.02)	607 (61.69)	< 0.001
MHR, median (IQR)	85.42 (74.15–98.95)	82.00 (76.11–89.45)	84.21 (74.06–94.89)	89.48 (78.06–102.00)	< 0.001
MBP, median (IQR)	76.79 (70.70–84.14)	74.45 (70.53–78.72)	75.69 (71.22–82.76)	73.64 (67.44–80.04)	< 0.001
MRR, median (IQR)	19.99 (17.55–22.96)	17.46 (15.81–19.30)	19.17 (17.01–22.42)	18.65 (16.38–21.80)	< 0.001
Temperature, median (IQR)	36.86 (36.65–37.19)	36.76 (36.53–37.05)	36.84 (36.62–37.09)	36.75 (36.51–37.00)	< 0.001
Spo2 mean, median (IQR)	96.71 (95.24–98.17)	97.90 (96.85–98.81)	96.84 (95.24–98.26)	97.05 (95.48–98.64)	< 0.001
Glu mean, median (IQR)	134.13 (109.53–170.48)	128.23 (119.88–139.60)	134.33 (113.00–166.43)	129.40 (107.67–160.92)	< 0.001
Myocardial infarct, n (%)	281 (20.24)	945 (20.64)	51 (24.06)	73 (7.42)	< 0.001
Congestive heart failure, n (%)	476 (34.29)	1134 (24.77)	85 (40.09)	152 (15.45)	< 0.001
Dementia, n (%)	143 (10.30)	73 (1.59)	12 (5.66)	13 (1.32)	< 0.001
Cerebrovascular disease, n (%)	219 (15.78)	545 (11.90)	34 (16.04)	67 (6.81)	< 0.001
Chronic pulmonary disease, n (%)	371 (26.73)	1056 (23.06)	52 (24.53)	218 (22.15)	0.024
Peptic ulcer disease, n (%)	45 (3.24)	48 (1.05)	4 (1.89)	95 (9.65)	< 0.001
Paraplegia, n (%)	78 (5.62)	122 (2.66)	12 (5.66)	18 (1.83)	< 0.001
Renal disease, n (%)	369 (26.59)	697 (15.22)	62 (29.25)	191 (19.41)	< 0.001
Malignant cancer, n (%)	259 (18.66)	295 (6.44)	35 (16.51)	126 (12.80)	< 0.001
Liver disease, n (%)	152 (10.95)	278 (6.07)	16 (7.55)	899 (91.36)	< 0.001
Diabetes, n (%)	443 (31.92)	1431 (31.25)	76 (35.85)	277 (28.15)	0.082
Constipation, n (%)	91 (6.56)	110 (2.40)	25 (11.79)	35 (3.56)	< 0.001
Hypertension, n (%)	457 (32.93)	2623 (57.28)	73 (34.43)	282 (28.66)	< 0.001
Obesity, n (%)	140 (10.09)	664 (14.50)	37 (17.45)	109 (11.08)	< 0.001
Charlson index, median (IQR)	6.00 (4.00–9.00)	5.00 (4.00–6.00)	6.00 (4.00–8.00)	6.00 (5.00–8.00)	< 0.001
APS III, median (IQR)	50.00 (39.00–66.00)	37.00 (28.00–53.00)	52.50 (36.00–68.00)	76.00 (58.00–97.25)	< 0.001
SAPS II, median (IQR)	40.00 (31.00–49.00)	35.00 (28.00–43.00)	39.00 (30.00–51.00)	45.00 (36.00–56.00)	< 0.001
OASIS, median (IQR)	34.00 (28.00–40.00)	32.00 (26.00–38.00)	33.00 (27.00–38.25)	38.00 (31.00–45.00)	< 0.001
SOFA, median (IQR)	5.00 (3.00–7.00)	5.00 (4.00–7.00)	5.00 (3.00–7.00)	9.00 (7.00–12.00)	< 0.001

*MHR* Mean heart rate, *MBP* Mean arterial blood pressure, *MRR* Mean respiratory rate, *Glu* Glucose, *APS III* Acute Physiology Score III, *SAPS I*I Simplified Acute Physiology Score II, *OASIS* Oxford Acute Severity of Illness Score, *SOFA* Sequential Organ Failure Assessment

P represent overall differences between all groups

Vital signs showed notable variations. The Lactulose group had the highest median heart rate (89.48 bpm, IQR: 78.06–102.00) and the lowest mean blood pressure (73.64 mmHg, IQR: 67.44–80.04). In contrast, the Senna group exhibited the highest respiratory rate (19.99 breaths/min, IQR: 17.55–22.96) and mean blood pressure (76.79 mmHg, IQR: 70.70–84.14).

The Lactulose group had the highest prevalence of liver disease (91.36%) but the lowest prevalence of myocardial infarction (7.42%). Conversely, the Docusate Sodium group had the lowest prevalence of liver disease (6.07%) and the highest prevalence of hypertension (57.28%). The Senna group exhibited the highest prevalence of dementia (10.30%), while the PEG group had the highest prevalence of congestive heart failure (40.09%), indicating a potentially higher cardiovascular disease burden in this group.

Using a 1:1 PSM algorithm, 1157 matched pairs of ICU patients receiving Docusate Sodium and Senna were identified. All baseline characteristics demonstrated SMD below 0.1, indicating excellent balance between the groups. (Supplementary Table 5).

### Survival analysis

**Fig. 2:**
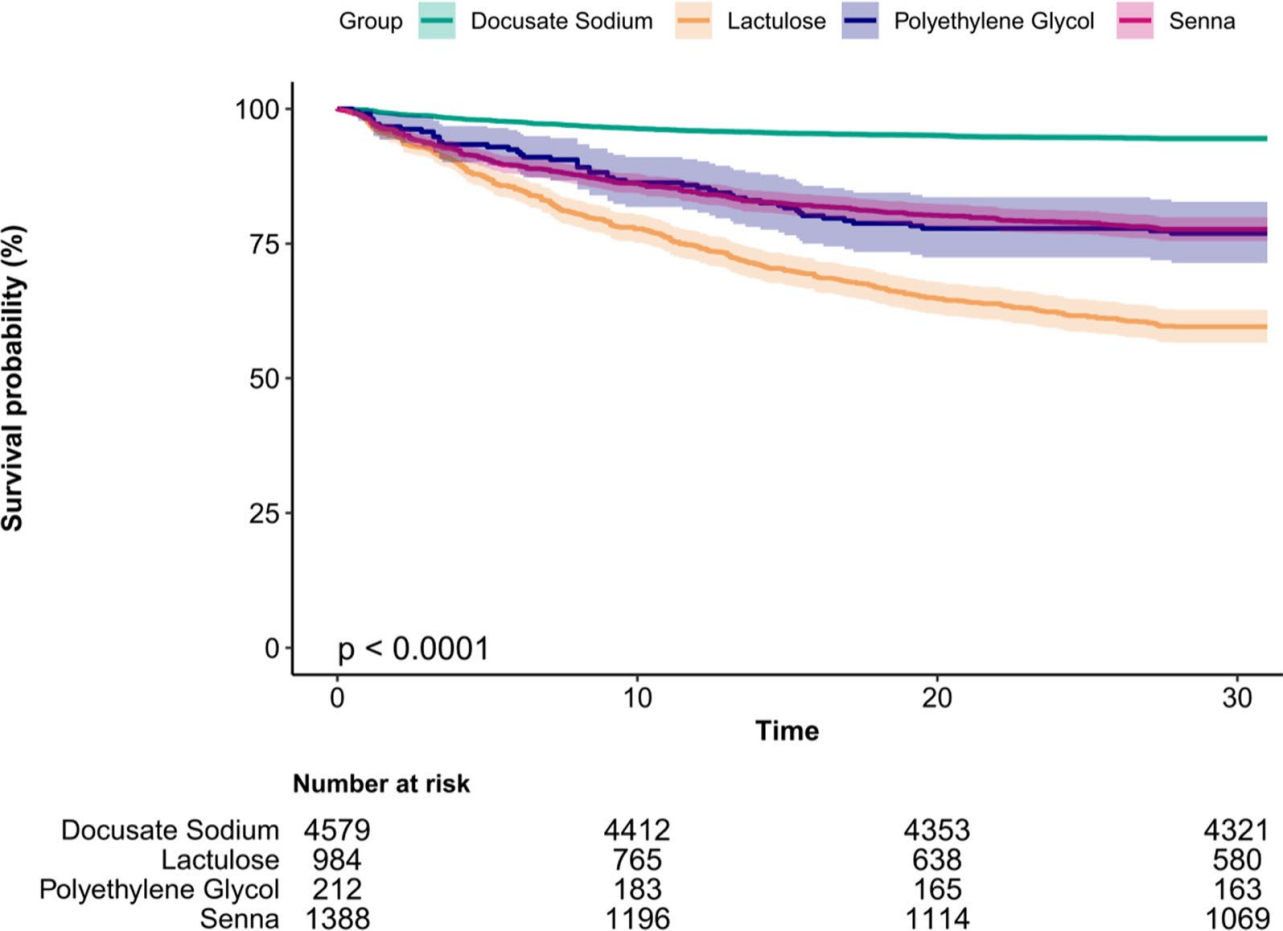
Kaplan–Meier survival curves for critically ill patients with sepsis receiving different bowel management treatments (Docusate Sodium, Lactulose, Polyethylene Glycol, and Senna). The survival probability (%) over time is shown for each treatment group, with shaded areas indicating 95% confidence intervals. Among the four groups, Docusate Sodium was associated with the highest survival probability, while Lactulose showed the lowest. Polyethylene Glycol and Senna displayed intermediate survival probabilities, with similar trends. Statistical analysis using the log-rank test demonstrated a highly significant difference among the groups (P < 0.001). The table below the graph shows the number of patients at risk at different time points

The Kaplan–Meier survival analysis revealed significant differences in survival probabilities among the four treatment groups (P < 0.001) (Fig. 2). Patients treated with Docusate Sodium demonstrated the highest survival probability and the slowest decline over time, suggesting a potential protective effect in this critically ill population. Conversely, the Lactulose group exhibited the lowest survival probability, indicating a potentially detrimental effect. Survival probabilities for the Polyethylene Glycol and Senna groups were intermediate and followed similar trends, though both were significantly lower compared to the Docusate Sodium group. The 95% confidence intervals (shaded areas) supported the robustness of these findings. These results underscore the influence of bowel management strategies on survival outcomes in critically ill septic patients.

### 28-day mortality

#### Preliminary findings: laxative use versus non-use

In the preliminary analysis, patients who received laxative treatment had significantly lower 28-day mortality compared to those who did not receive laxatives [adjusted hazard ratio (HR): 0.57; 95% CI 0.53–0.62; P < 0.001]. Full results of this comparison are provided in Supplementary Tables 1–4.

#### Primary analysis: comparison among laxative agents

This study assessed the association between laxative use and 28-day mortality in ICU patients with sepsis. Compared to the reference group receiving Senna, Docusate Sodium was associated with a significantly lower risk of 28-day mortality across all models. In the crude model, the HR was 0.22 (95% CI 0.19–0.26, P < 0.001), and after full adjustment in Model 3, the HR remained significantly reduced at 0.43 (95% CI 0.36–0.52, P < 0.001). These findings suggest that Docusate Sodium may be a safer and potentially beneficial option for bowel management in critically ill septic patients (Table 2).

**Table 2:** Association between laxative use and 28-day mortality among ICU patients with sepsis

Laxatives	HR (95% CI)									
	No. total	No. event%	Crude	P	Model1	P	Model2	P	Model3	P
Senna	1388	310 (22.3)	1(Ref)		1(Ref)		1(Ref)		1(Ref)	
Docusate Sodium	4579	252 (5.5)	0.22 (0.19–0.26)	< 0.001	0.24 (0.21–0.29)	< 0.001	0.35 (0.29–0.42)	< 0.001	0.43 (0.36–0.52)	< 0.001
Polyethylene Glycol (PEG)	212	49 (23.1)	1.03 (0.76–1.39)	0.854	1.04 (0.77–1.40)	0.807	1.05 (0.77–1.43)	0.778	1.06 (0.78–1.45)	0.701
Lactulose	984	398 (40.4)	1.98 (1.71–2.30)	< 0.001	2.69 (2.29–3.16)	< 0.001	2.60 (2.19–3.09)	< 0.001	1.82 (1.45–2.27)	< 0.001
P for trend				< 0.001		< 0.001		< 0.001		< 0.001

This table presents the association between laxative use and 28-day mortality in ICU patients with sepsis. Hazard ratios (HR) and 95% confidence intervals (CI) are reported for each laxative group, with Senna as the reference. Crude HRs (unadjusted), Model 1, Model 2, and adjusted HRs (Model 3) are displayed. Model 1 adjusts for age, race, and gender. Model 2 further adjusts for vital signs, including mean heart rate, mean blood pressure, mean respiratory rate, mean temperature, mean SpO₂, and mean glucose. Model 3 incorporates additional adjustments for comorbidities, including myocardial infarction, dementia, cerebrovascular disease, chronic pulmonary disease, peptic ulcer disease, liver disease, diabetes, paraplegia, renal disease, obesity, malignant cancer, hypertension, and congestive heart failure. Statistical significance is defined as P < 0.05, with P for trend < 0.001 indicating significant differences among laxatives

Conversely, Lactulose was associated with a significantly higher risk of 28-day mortality compared to Senna. The crude HR was 1.98 (95% CI 1.71–2.30, P < 0.001), and the adjusted HR in Model 3 was 1.82 (95% CI 1.45–2.27, P < 0.001). The markedly higher prevalence of liver disease in the Lactulose group (91.36%) may partly explain this association, as underlying disease severity and Lactulose’s hyperosmotic effects could exacerbate outcomes. These findings suggest careful evaluation is needed when considering Lactulose in this population.

The analysis assessed the association between Senna and Docusate Sodium use and 28-day mortality among ICU sepsis patients using propensity score adjustment and matching. Docusate Sodium significantly reduced the odds of 28-day mortality compared to Senna in both models (adjusted OR: 0.49, 95% CI 0.40–0.60, P < 0.001; matched OR: 0.60, 95% CI 0.49–0.74, P < 0.001) (Supplementary Table 6).

### Secondary outcomes

This study evaluated the association between laxative use and various secondary outcomes, including bowel sounds recovery, the risk of C-diff infection, ICU-free days, vasopressor-free days, and ventilator-free days in ICU patients with sepsis.

### Bowel sounds conversion

Docusate Sodium significantly increased the likelihood of bowel sounds recovery compared to Senna (adjusted OR: 2.70, 95% CI 2.30–3.16, P < 0.001), suggesting its potential benefit in promoting gut motility in critically ill patients. Polyethylene Glycol (PEG) showed no significant effect (adjusted OR: 0.94, 95% CI 0.65–1.36, P = 0.755), indicating a neutral impact. Lactulose slightly increased recovery odds compared to Senna (adjusted OR: 1.12, 95% CI 0.86–1.47, P = 0.409), but the effect was not statistically significant. These findings support the use of Docusate Sodium as an effective option for bowel management in septic ICU patients (Table 3).

**Table 3:** Association between laxative use and bowel sounds conversion among ICU patients with sepsis (Model 3)

Laxatives	OR (95% CI)					
	No. total	No. event%	Crude	P value	Model3	P value
Senna	1388	296 (21.3)	1(Ref)		1(Ref)	
Docusate Sodium	4579	2517 (55.0)	4.50 (3.91–5.19)	< 0.001	2.70 (2.30–3.16)	< 0.001
Polyethylene Glycol (PEG)	212	47 (22.2)	1.05 (0.74–1.49)	0.78	0.94 (0.65–1.36)	0.755
Lactulose	984	236 (24.0)	1.16 (0.96–1.41)	0.126	1.12 (0.86–1.47)	0.409
P for trend				0.001		0.063

This table examines the association between laxative use and bowel sounds conversion in ICU patients with sepsis. Odds ratios (OR) and 95% confidence intervals (CI) are provided for each laxative group, using Senna as the reference. Crude and adjusted ORs (Model 3) are shown. Model 3 adjusts for age, race, gender, mean heart rate, mean blood pressure, mean respiratory rate, mean temperature, mean SpO₂, mean glucose, myocardial infarction, dementia, cerebrovascular disease, chronic pulmonary disease, peptic ulcer disease, liver disease, diabetes, paraplegia, renal disease, obesity, malignant cancer, hypertension, and congestive heart failure. A P for trend indicates the significance of overall differences between groups

### Risk of clostridium difficile infection

Docusate Sodium (adjusted OR: 0.60, 95% CI 0.29–1.24, P = 0.165) and PEG (adjusted OR: 0.62, 95% CI 0.08–4.80, P = 0.643) showed no significant association with the risk of C-diff infection compared to Senna. In contrast, Lactulose significantly increased the risk of C-diff infection (adjusted OR: 2.67, 95% CI 1.23–5.83, P = 0.013), indicating a 167% higher risk. The P for trend was < 0.001, highlighting significant differences among laxative types. These findings suggest that while Docusate Sodium and PEG are relatively safe, Lactulose may increase C-diff risk (Table 4).

**Table 4:** Association between laxative use and C-diff infection among ICU patients with sepsis (Model 1)

Laxatives	OR (95% CI)					
	No. total	No. event%	Crude	P value	Model 1	P value
Senna	1388	11 (0.8)	1(Ref)		1(Ref)	
Docusate Sodium	4579	23 (0.5)	0.62 (0.30–1.27)	0.191	0.60 (0.29–1.24)	0.165
Polyethylene Glycol (PEG)	212	1 (0.5)	0.62 (0.08–4.87)	0.653	0.62 (0.08–4.80)	0.643
Lactulose	984	20 (2.0)	2.45 (1.17–5.14)	0.018	2.67 (1.23–5.83)	0.013
P for trend				< 0.001		< 0.001

This table shows the association between different laxatives and the risk of Clostridium difficile (C-diff) infection in ICU patients with sepsis. Odds ratios (OR) and 95% confidence intervals (CI) are reported for each laxative group, with Senna as the reference. Crude ORs and adjusted ORs (Model 1) are provided. Model 1 adjusts for age, race, and gender. Statistical significance is indicated, with P < 0.05 considered significant. The P for trend reflects significant variability in the risk of C-diff infection across the laxative groups, with a P value < 0.001 indicating significant differences

### ICU-free days

Docusate Sodium significantly increased ICU-free days compared to Senna (adjusted mean difference: 2.07 days, 95% CI 1.50–2.65, P < 0.001), suggesting a benefit in reducing ICU dependence. In contrast, PEG slightly reduced ICU-free days by 1.41 days (95% CI −2.65 to −0.18, P = 0.025), and Lactulose significantly reduced ICU-free days by 5.95 days (95% CI −6.91 to −5.00, P < 0.001), indicating increased ICU dependency. The P for trend was < 0.001, underscoring significant differences among laxatives (Table 5).

**Table 5:** Association between laxative use and ICU-free days, Vasopressor-free days, and ventilator-free days within 28 days among ICU patients with sepsis

Laxatives	ICU-free days	Crude	P	Model 3	P	Vasopressor-free Days	Crude	P	Model 3	P	ventilator-free days	Crude	P	Model 3	P
Senna	24.35 (15.46–26.09)	0 (Ref)		0 (Ref)		28.00 (23.49–28.00)	0 (Ref)		0 (Ref)		27.48 (21.44–28.00)	0 (Ref)		0 (Ref)	
Docusate Sodium	25.88 (24.04–26.71)	4.67 (4.14–5.19)	< 0.001	2.07 (1.50–2.65)	< 0.001	27.77 (27.01–27.99)	4.35 (3.81–4.90)	< 0.001	1.96 (1.36–2.57)	< 0.001	27.65 (27.15–27.83)	4.51 (3.96–5.05)	< 0.001	1.96 (1.35–2.57)	< 0.001
Polyethylene Glycol (PEG)	23.36 (8.14–25.73)	−1.17 (−2.44–0.09)	0.069	−1.41 (−2.65–0.18)	0.025	27.78 (16.88–28.00)	−0.57 (−1.88–0.75)	0.401	−0.65 (−1.95–0.65)	0.328	27.57 (18.75–28.00)	−0.37 (−1.69–0.94)	0.578	−0.52 (−1.83–0.79)	0.433
Lactulose	16.72 (0.00–24.78)	−6.06 (−6.78− −5.35)	< 0.001	−5.95 (−6.91− −5.00)	< 0.001	24.74 (0.00–28.00)	−5.74 (−6.48− −4.99)	< 0.001	−5.35 (−6.35–4.34)	< 0.001	23.50 (0.00–28.00)	−5.7 (−6.44− −4.96)	< 0.001	−5.53 (−6.54− −4.51)	< 0.001
P for trend			< 0.001		< 0.001			< 0.001		< 0.001			< 0.001		< 0.001

This table presents the association between different laxatives and three key outcomes within 28 days among ICU patients with sepsis: ICU-free days, vasopressor-free days, and ventilator-free days. Mean days (95% CI) are reported for each laxative group, along with crude and adjusted mean differences (Model 3), using Senna as the reference group. Model 3 adjusts for age, race, gender, mean heart rate, mean blood pressure, mean respiratory rate, mean temperature, mean SpO₂, mean glucose, and comorbidities, including myocardial infarction, dementia, cerebrovascular disease, chronic pulmonary disease, peptic ulcer disease, liver disease, diabetes, paraplegia, renal disease, obesity, malignant cancer, hypertension, and congestive heart failure. P for trend indicates overall differences among laxative groups, with P < 0.05 considered statistically significant

### Vasopressor-free days

Compared to Senna, Docusate Sodium was associated with a slight reduction in vasopressor-free days (adjusted mean difference: −1.96 days, 95% CI −2.57 to −1.36, P < 0.001), but the effect was minimal, suggesting relative safety. PEG showed no significant effect (−0.65 days, 95% CI −1.95 to 0.65, P = 0.328), indicating a neutral impact. Lactulose significantly reduced vasopressor-free days by 5.35 days (95% CI −6.35 to −4.34, P < 0.001), indicating increased vasopressor dependence and potential cardiovascular instability. The P for trend was < 0.001, showing significant differences among laxative groups (Table 5).

### Ventilator-free days

Docusate Sodium significantly increased ventilator-free days compared to Senna (adjusted mean difference: 1.96 days, 95% CI 1.35–2.57, P < 0.001), while PEG had no significant effect (−0.52 days, 95% CI −1.83 to 0.79, P = 0.433). Lactulose significantly reduced ventilator-free days by 5.53 days (95% CI −6.54 to −4.51, P < 0.001), indicating increased mechanical ventilation dependency. The P for trend was < 0.001, highlighting significant differences across groups (Table 5).

### Subgroup analysis of mortality

**Fig. 3:**
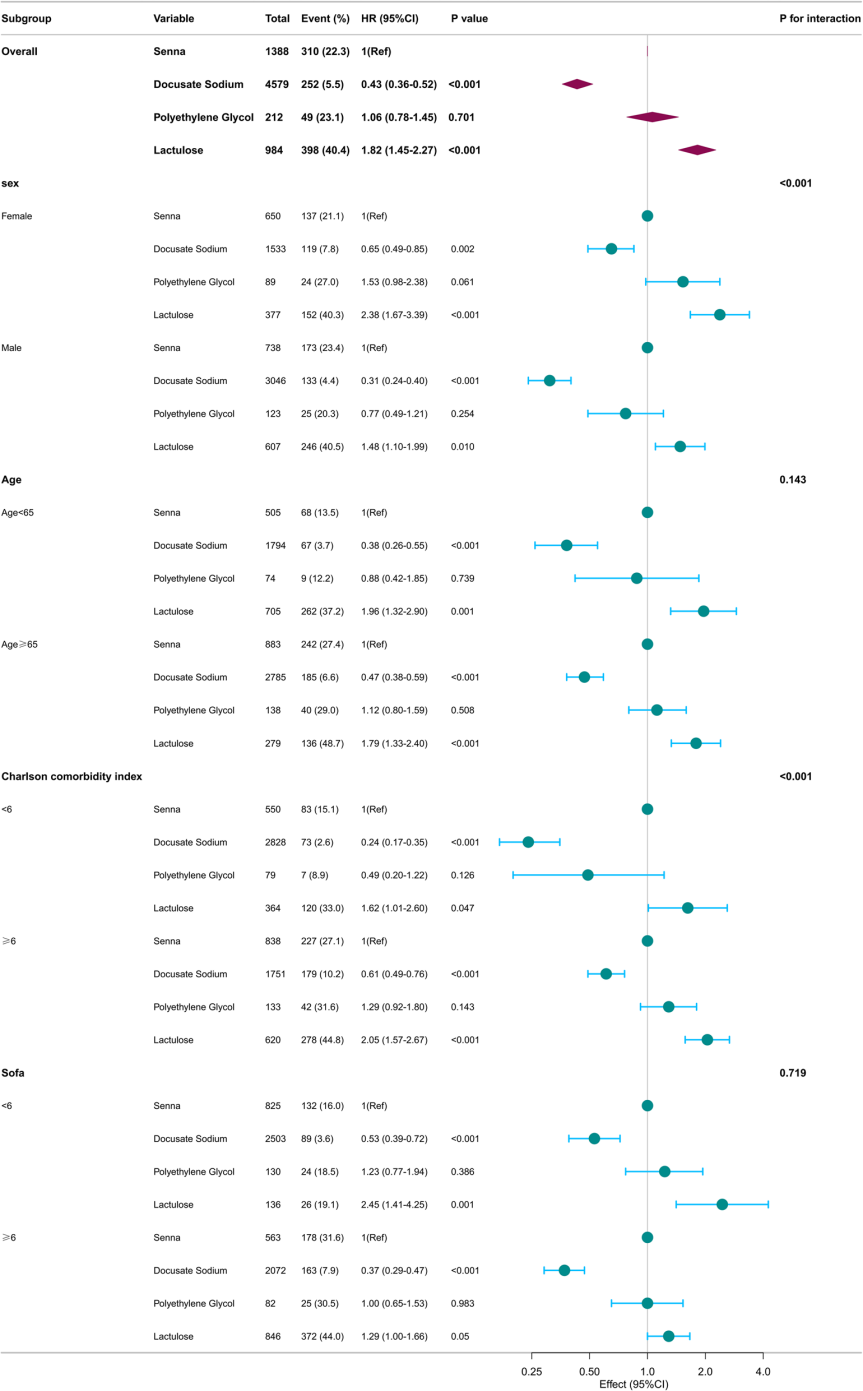
Subgroup analysis of 28-day mortality by laxative type among ICU patients with sepsis. This forest plot illustrates the hazard ratios (HRs) and 95% confidence intervals (CIs) for 28-day mortality in various subgroups of ICU patients with sepsis treated with different laxatives. Subgroups include stratifications by sex (female and male), age (< 65 and > 65 years), Charlson Comorbidity Index (CCI < 6 and > 6), and SOFA score (< 6 and > 6). Senna is the reference group (HR = 1). Hazard ratios below 1 indicate reduced mortality risk, while values above 1 suggest increased mortality risk. The interaction P-values evaluate whether the effect of laxatives on mortality differs across subgroups. Significant interactions highlight variability in treatment effects, emphasizing the importance of personalized therapy in critically ill patients

Subgroup analysis showed that Docusate Sodium significantly reduced 28-day mortality in ICU sepsis patients across sex, age, CCI, and SOFA score subgroups (Fig. 3). Its protective effect was stronger in males (HR: 0.31, 95% CI 0.24–0.40) and patients with fewer comorbidities (CCI < 6: HR: 0.24, 95% CI 0.17–0.35). Lactulose, in contrast, consistently increased mortality, with the highest risk observed in females (HR: 2.38, 95% CI 1.67–3.39) and patients with severe comorbidities (CCI ≥ 6: HR: 2.05, 95% CI 1.57–2.67). No significant interactions were observed for age (P = 0.14) or SOFA score (P = 0.72). These findings suggest that Docusate Sodium is a safer option for bowel management, particularly in vulnerable populations.

## Discussion

Our preliminary comparison showed that laxative use was associated with significantly reduced 28-day mortality among ICU septic patients, consistent with prior studies suggesting potential benefits of early bowel management in critical illness [9]. However, this finding should be interpreted with caution due to the considerable heterogeneity of the non-laxative group, which likely included patients with early mortality, shorter ICU stays, or contraindications to laxative administration.

Our results indicate that Docusate Sodium use was associated with a significantly lower risk of 28-day mortality compared with Senna (adjusted HR = 0.43; 95% CI 0.36–0.52). In contrast, Lactulose use was linked to a notably higher risk of mortality (adjusted HR = 1.82; 95% CI 1.45–2.27). It is important to note that most patients receiving Lactulose had underlying liver disease, implying different therapeutic goals for this subgroup. This clinical heterogeneity should be accounted for when interpreting our findings.

With regard to secondary outcomes, patients on Docusate Sodium had more ICU-free days and faster recovery of bowel sounds compared to Senna, whereas those on Lactulose experienced fewer ICU-free days and a higher incidence of C-diff infection. Taken together, these observations underscore the potential survival benefits of Docusate Sodium for bowel management in critically ill sepsis patients.

Our findings are consistent with earlier studies on laxative use in critically ill populations. For example, Tao H et al. [9] demonstrated that Docusate Sodium not only improved bowel function but was also associated with reduced mortality (n = 253; odds ratio [OR] = 0.59; 95% CI 0.42–0.83; P = 0.002). Like our study, Tao H et al. adjusted for multiple confounders, including age, sex, comorbidities, and illness severity scores, reinforcing the robustness of both sets of results. Notably, their work suggested that the benefits of Docusate might be more pronounced in more acutely ill patients without COPD, diabetes, or renal disease.

Despite the general agreement in primary outcomes, slight differences emerged in subgroup analyses. These discrepancies may be attributable to methodological and population-related factors. Tao et al. focused primarily on mechanically ventilated patients and used a relatively small sample, whereas we analyzed a larger cohort of septic patients. Additionally, our use of propensity score matching to balance baseline characteristics likely strengthened causal inference beyond what can be achieved through standard multivariable adjustments alone.

Our observations regarding Lactulose differ from those of Odenwald et al. [15], who investigated Lactulose exclusively in patients with chronic liver disease and found increased densities of intestinal bifidobacteria alongside reduced rates of systemic infections and mortality. This discrepancy likely stems from differences in the populations studied. Because Odenwald et al. focused solely on patients with chronic liver disease, Lactulose’s principal benefit—lowering ammonia levels—may not translate to a general ICU population. In non-hepatic critically ill patients, Lactulose’s osmotic effects could potentially exacerbate fluid and electrolyte imbalances, leading to worse outcomes [16]. Although Lactulose use was associated with increased mortality and C-diff infection risk, this finding should be interpreted cautiously due to the high prevalence of liver disease in this subgroup. The results may reflect underlying clinical complexity rather than a direct adverse effect of the drug.

Our findings also align with mechanistic perspectives on how laxatives operate in critically ill patients. Docusate Sodium, a stool softener, promotes bowel motility while exerting minimal effects on fluid and electrolyte balance, which may underlie its association with better outcomes [17]. These observations highlight the importance of individualizing laxative therapy in critically ill settings.

To further clarify the impact of patient characteristics on our main findings, we conducted subgroup analyses examining potential effect modifications by sex, age, CCI, and SOFA score. Notably, there was a significant interaction for sex and comorbidity burden: Docusate Sodium’s protective effect was more pronounced in male patients and those with fewer comorbidities, whereas Lactulose was associated with higher mortality in female patients and those with a greater comorbidity burden. We did not detect significant interactions with age or SOFA score, suggesting that the overall findings are broadly applicable across various levels of illness severity.

Several features of our study bolster its clinical relevance. First, leveraging the MIMIC-IV database provided access to granular clinical data on ICU admissions, allowing for a large-scale, in-depth analysis of critically ill sepsis patients. The inclusion of 7163 patients yielded sufficient statistical power to detect meaningful associations between laxative use and clinical outcomes. Second, our rigorous design—featuring propensity score matching—minimized baseline imbalances across treatment groups, enhancing the validity of our findings. Third, using multiple adjusted models (Models 1, 2, and 3) facilitated a stepwise exploration of how demographic, physiological, and comorbidity-related variables influenced outcomes. Finally, by focusing on critically ill patients and delving into secondary outcomes, our conclusions are directly applicable to ICU practice.

## Limitation

Despite these strengths, this study has several limitations. First, reliance on a single-center database (MIMIC-IV) from the United States may limit the generalizability of our findings to other healthcare systems or geographic regions. Second, our exclusion criteria (e.g., concurrent use of multiple laxatives, ICU stays under 24 h) may reduce the applicability of the results to broader ICU populations. Third, although propensity score matching helps control for confounders, we performed PSM on only the two groups with the largest sample sizes due to group sample size balance issues, potentially influencing the differences in results and limiting causality assessment within our observational design. Lastly, our cohort consisted predominantly of critically ill sepsis patients, restricting extrapolation to other patient populations or clinical severities.

Future research should include multicenter, prospective studies encompassing diverse patient populations, clearly defined control groups, and possibly IPTW or stabilized weighting methods, thereby enhancing generalizability and causal interpretation of outcomes associated with laxative use in critically ill patients.

## Conclusion

Docusate sodium appears to be a safer and more effective bowel management option for critically ill patients with sepsis. In contrast, the association between lactulose use and adverse outcomes may primarily reflect the severity of underlying liver disease rather than a direct drug effect. These findings underscore the importance of individualized laxative selection based on patients’ clinical context in critical care practice.

## Supplementary Information

Additional file 1.

## Data Availability

The data was extracted from Medical Information Mart for Intensive Care IV (MIMIC-IV, Version 3.1). The identification information was concealed and privacy of patients in MIMIC-IV were protected. Thus, there were no additional consent procedures from the institutional ethics committee.

## Abbreviations

ICUs: Intensive care units
STROBE: Strengthening the Reporting of Observational Studies in Epidemiology
SOFA: Sequential Organ Failure Assessment
PEG: Polyethylene Glycol
CCI: Charlson Comorbidity Index
APS III: Acute Physiology Score III
SAPS II: Simplified Acute Physiology Score II
OASIS: Oxford Acute Severity of Illness Score
IQRs: Interquartile ranges
MAP: Mean arterial pressure
C-diff: Clostridium difficile
ORs: Odds ratios
CIs: Confidence intervals
PSM: Propensity score matching
SMD: Standardized mean difference
HR: Hazard ratio
