# Supplementary material for: Laxative use and 28-day mortality in critically ill sepsis patients: a retrospective cohort study using MIMIC-IV (v3.1)
Source: J Intensive Care. 2025 May 21;13:27. doi: 10.1186/s40560-025-00797-9 (PMC12093854; doi:10.1186/s40560-025-00797-9)
Supplement: Supplementary file 1 — Additional file 1. [file 40560_2025_797_MOESM1_ESM.docx]

**Supplementary Table 1. Baseline characteristics of ICU patients Non-laxative vs. Laxative.**

| **Variables** | **Total (n = 14300)** | **Non-laxative**  **(n = 7137)** | **Laxative**  **(n = 7163)** | **P** |
| --- | --- | --- | --- | --- |
| **Age, Median (IQR)** | 67.8 (57.0, 78.4) | 68.2 (56.0, 79.7) | 67.5 (57.9, 77.0) | 0.013 |
| **Race White, n (%)** | 9723 (68.0) | 4700 (65.9) | 5023 (70.1) | < 0.001 |
| **Male, n (%)** | 8409 (58.8) | 3895 (54.6) | 4514 (63) | < 0.001 |
| **MHR, Median (IQR)** | 85.4 (76.3, 97.1) | 88.7 (77.0, 100.8) | 83.0 (75.9, 92.7) | < 0.001 |
| **MBP, Median (IQR)** | 75.0 (69.9, 81.2) | 75.4 (69.6, 82.7) | 74.7 (70.2, 79.9) | < 0.001 |
| **MRR, Median (IQR)** | 18.7 (16.5, 21.6) | 19.6 (17.1, 22.6) | 18.0 (16.1, 20.5) | < 0.001 |
| **Temperature, Median (IQR)** | 36.8 (36.6, 37.1) | 36.9 (36.6, 37.2) | 36.8 (36.6, 37.1) | < 0.001 |
| **Spo2 mean, Median (IQR)** | 97.4 (96.0, 98.6) | 97.1 (95.7, 98.4) | 97.6 (96.3, 98.7) | < 0.001 |
| **Glu mean, Median (IQR)** | 130.2 (115.0, 155.2) | 133.0 (110.5, 166.3) | 128.9 (117.8, 146.0) | < 0.001 |
| **Myocardial infarct, n (%)** | 2353 (16.5) | 1003 (14.1) | 1350 (18.8) | < 0.001 |
| **Congestive heart failure, n (%)** | 3723 (26.0) | 1876 (26.3) | 1847 (25.8) | 0.495 |
| **Peripheral vascular disease, n (%)** | 1796 (12.6) | 859 (12) | 937 (13.1) | 0.059 |
| **cerebrovascular_disease, n (%)** | 1619 (11.3) | 754 (10.6) | 865 (12.1) | 0.004 |
| **Chronic pulmonary disease, n (%)** | 3451 (24.1) | 1754 (24.6) | 1697 (23.7) | 0.216 |
| **Rheumatic disease, n (%)** | 486 ( 3.4) | 247 (3.5) | 239 (3.3) | 0.682 |
| **Peptic ulcer disease, n (%)** | 566 ( 4.0) | 374 (5.2) | 192 (2.7) | < 0.001 |
| **Paraplegia, n (%)** | 487 ( 3.4) | 257 (3.6) | 230 (3.2) | 0.199 |
| **Liver disease, n (%)** | 2391 (16.7) | 1046 (14.7) | 1345 (18.8) | < 0.001 |
| **Diabetes, n (%)** | 4344 (30.4) | 2117 (29.7) | 2227 (31.1) | 0.063 |
| **Hypertension, n (%)** | 6176 (43.2) | 2741 (38.4) | 3435 (48) | < 0.001 |
| **Obesity, n (%)** | 1692 (11.8) | 742 (10.4) | 950 (13.3) | < 0.001 |
| **Renal disease, n (%)** | 2967 (20.7) | 1648 (23.1) | 1319 (18.4) | < 0.001 |
| **Malignant cancer, n (%)** | 2034 (14.2) | 1319 (18.5) | 715 (10) | < 0.001 |
| **Charlson index, Median (IQR)** | 6.0 (4.0, 8.0) | 6.0 (4.0, 8.0) | 5.0 (4.0, 7.0) | < 0.001 |
| **Aps III, Median (IQR)** | 48.0 (35.0, 68.0) | 52.0 (39.0, 70.0) | 44.0 (32.0, 66.0) | < 0.001 |
| **Saps II, Median (IQR)** | 38.0 (30.0, 49.0) | 39.0 (31.0, 50.0) | 37.0 (30.0, 47.0) | < 0.001 |
| **OASIS, Median (IQR)** | 34.0 (28.0, 40.0) | 34.0 (28.0, 41.0) | 33.0 (27.0, 39.0) | < 0.001 |
| **SOFA, Median (IQR)** | 5.0 (4.0, 8.0) | 5.0 (3.0, 8.0) | 5.0 (4.0, 8.0) | < 0.001 |

**Note:** MHR: Mean heart rate; MBP: Mean arterial blood pressure; MRR: Mean respiratory rate; Glu: Glucose; APS III: Acute Physiology Score III; SAPS II: Simplified Acute Physiology Score II, OASIS: Oxford Acute Severity of Illness Score, and SOFA: Sequential Organ Failure Assessment. P represent overall differences between all groups.

**Supplementary Table 2. Relationship between Laxative and 28-day mortality (Cox).**

| **Variable** | **HR (95% CI)** | | | | | |
| --- | --- | --- | --- | --- | --- | --- |
|  | **No.total** | **No.event%** | **Crude** | **P** | **Model3** | **P** |
| **Non-laxative** | 7137 | 1636 (22.9) | 1(Ref) |  | 1(Ref) |  |
| **Laxative** | 7163 | 1009 (14.1) | 0.57 (0.53-0.62) | <0.001 | 0.57 (0.53-0.62) | <0.001 |

**Note:** This table presents the association between laxative use and 28-day mortality in ICU patients with sepsis. Hazard ratios (HR) and 95% confidence intervals (CI) are reported for each laxative group, with Non-laxative as the reference. Crude HRs (unadjusted) and adjusted HRs (Model 3) are displayed. Model 3 further adjusts for age, race, and gender, vital signs, including mean heart rate, mean blood pressure, mean respiratory rate, mean temperature, mean SpO₂, and mean glucose, incorporates additional adjustments for comorbidities, including myocardial infarction, dementia, cerebrovascular disease, chronic pulmonary disease, peptic ulcer disease, liver disease, diabetes, paraplegia, renal disease, obesity, malignant cancer, hypertension, and congestive heart failure. Statistical significance is defined as P < 0.05, with P for trend <0.001 indicating significant differences two groups.

**Supplementary Table 3. Relationship between Laxative and Secondary Outcomes (Logistic).**

| **Variable** | **OR (95% CI)** | | | | | | | |
| --- | --- | --- | --- | --- | --- | --- | --- | --- |
|  | **No.total** | **No.event%** | **Crude** | **P** | **Model1** | **P** | **Model3** | **P** |
| **Bowel Sounds Conversion** | | | | | | | | |
| **Non-laxative** | 7137 | 1731 (24.3) | 1(Ref) |  | - | - | 1(Ref) |  |
| **Laxative** | 7163 | 3096 (43.2) | 2.38 (2.21-2.55) | <0.001 | - | - | 1.89 (1.74-2.04) | <0.001 |
| **C-diff Infection** | | | | | | | | |
| **Non-laxative** | 7137 | 112 (1.6) | 1(Ref) |  | 1(Ref) |  | - | - |
| **Laxative** | 7163 | 55 (0.8) | 0.49 (0.35-0.67) | <0.001 | 0.55 (0.39-0.78) | <0.001 | - | - |

**Note:** This table presents the association between laxative use and Secondary Outcomes in ICU patients with sepsis. Odds ratios (OR) and 95% confidence intervals (CI) are reported for each laxative group, with Non-laxative as the reference. Crude ORs (unadjusted), Model 1 and adjusted ORs (Model 3) are displayed. Model 1 adjusts for age, race, and gender. Model 3 further adjusts for vital signs, including mean heart rate, mean blood pressure, mean respiratory rate, mean temperature, mean SpO₂, and mean glucose, incorporates additional adjustments for comorbidities, including myocardial infarction, dementia, cerebrovascular disease, chronic pulmonary disease, peptic ulcer disease, liver disease, diabetes, paraplegia, renal disease, obesity, malignant cancer, hypertension, and congestive heart failure. Statistical significance is defined as P < 0.05, with P for trend <0.001 indicating significant differences two groups.

**Supplementary Table 4. Relationship between Laxative and Secondary Outcomes (Linear Regression).**

| **Variable** | **OR (95% CI)** | | | | |
| --- | --- | --- | --- | --- | --- |
|  | **No.total** | **Crude** | **P** | **Model3** | **P** |
| **ICU-Free Days** | | | | | |
| **Non-laxative** | 7137 | 0(Ref) |  | 0(Ref) |  |
| **Laxative** | 7163 | 2.22 (1.87-2.58) | <0.001 | 0.87 (0.52-1.22) | ＞0.05 |
| **Vasopressor-Free Days** | | | | | |
| **Non-laxative** | 7137 | 0(Ref) |  | 0(Ref) |  |
| **Laxative** | 7163 | 2.22 (1.87-2.58) | <0.001 | 0.87 (0.52-1.22) | ＞0.05 |
| **Ventilator-Free Days** | | | | | |
| **Non-laxative** | 7137 | 0(Ref) |  | 0(Ref) |  |
| **Laxative** | 7163 | 2.36 (2-2.71) | <0.001 | 0.96 (0.61-1.31) | ＞0.05 |

**Note:** This table presents the association between laxative use and Secondary Outcomes in ICU patients with sepsis. Hazard ratios (HR) and 95% confidence intervals (CI) are reported for each laxative group, with Non-laxative as the reference. Crude HRs (unadjusted) and adjusted HRs (Model 3) are displayed. Model 3 further adjusts for age, race, and gender, vital signs, including mean heart rate, mean blood pressure, mean respiratory rate, mean temperature, mean SpO₂, and mean glucose, incorporates additional adjustments for comorbidities, including myocardial infarction, dementia, cerebrovascular disease, chronic pulmonary disease, peptic ulcer disease, liver disease, diabetes, paraplegia, renal disease, obesity, malignant cancer, hypertension, and congestive heart failure. Statistical significance is defined as P < 0.05, with P for trend <0.001 indicating significant differences two groups.

**Supplementary Table 5. Baseline characteristics of ICU patients by propensity-matched ICU patients Senna vs. Docusate Sodium.**

|  | **Before PSM** | | | **After PSM** | | |
| --- | --- | --- | --- | --- | --- | --- |
| **Characteristic** | **Senna**  **(n = 1,388)** | **Docusate Sodium**  **(n = 4,579)** | **SMD** | **Senna**  **(n = 1,157)** | **Docusate Sodium**  **(n = 1,157)** | **SMD** |
| **Male，n%** | 738.00 (53.17%) | 3,046.00 (66.52%) | 0.27 | 635.00 (54.88%) | 644.00 (55.66%) | 0.02 |
| **Age, Median (IQR)** | 71.09 (58.81- 82.41) | 68.49 (59.97-77.01) | 0.07 | 70.24 (57.44-81.60) | 67.49 (57.83- 78.51) | 0.04 |
| **Race White, n (%)** | 877.00 (63.18%) | 3,378.00 (73.77%) | 0.27 | 751.00 (64.91%) | 751.00 (64.91%) | 0.01 |
| **MHR, Median (IQR)** | 85.42 (74.14- 98.98) | 82.00 (76.11- 89.45) | 0.24 | 84.08 (73.83-97.00) | 83.54 (75.91- 94.10) | 0.00 |
| **MBP, Median (IQR)** | 76.79 (70.69- 84.15) | 74.45 (70.54-78.72) | 0.33 | 76.10 (70.38-83.68) | 76.38 (71.07-82.37) | 0.00 |
| **MRR, Median (IQR)** | 19.99 (17.55-22.96) | 17.46 (15.81-19.30) | 0.73 | 19.50 (17.21-22.29) | 19.00 (16.97-21.85) | 0.06 |
| **Temperature, Median (IQR)** | 36.86 (36.64-37.19) | 36.72 (36.44-36.99) | 0.34 | 36.85 (36.63-37.18) | 36.87 (36.55-37.20) | 0.01 |
| **Myocardial infarct, n (%)** | 281.00 (20.24%) | 945.00 (20.64%) | 0.01 | 231.00 (19.97%) | 213.00 (18.41%) | 0.04 |
| **Congestive heart failure, n (%)** | 476.00 (34.29%) | 1,134.00 (24.77%) | 0.21 | 385.00 (33.28%) | 375.00 (32.41%) | 0.02 |
| **dementia, n (%)** | 143.00 (10.30%) | 73.00 (1.59%) | 0.37 | 78.00 (6.74%) | 62.00 (5.36%) | 0.06 |
| **Cerebrovascular disease, n (%)** | 219.00 (15.78%) | 545.00 (11.90%) | 0.11 | 178.00 (15.38%) | 170.00 (14.69%) | 0.02 |
| **Chronic pulmonary disease, n (%)** | 371.00 (26.73%) | 1,056.00 (23.06%) | 0.08 | 311.00 (26.88%) | 300.00 (25.93%) | 0.02 |
| **Peptic ulcer disease, n (%)** | 45.00 (3.24%) | 48.00 (1.05%) | 0.15 | 33.00 (2.85%) | 35.00 (3.03%) | 0.01 |
| **Paraplegia, n (%)** | 78.00 (5.62%) | 122.00 (2.66%) | 0.15 | 58.00 (5.01%) | 61.00 (5.27%) | 0.01 |
| **Liver disease, n (%)** | 152.00 (10.95%) | 278.00 (6.07%) | 0.18 | 126.00 (10.89%) | 128.00 (11.06%) | 0.01 |
| **Diabetes, n (%)** | 443.00 (31.92%) | 1,431.00 (31.25%) | 0.01 | 363.00 (31.37%) | 353.00 (30.51%) | 0.02 |
| **Renal disease, n (%)** | 369.00 (26.59%) | 697.00 (15.22%) | 0.28 | 292.00 (25.24%) | 300.00 (25.93%) | 0.02 |
| **Malignant cancer, n (%)** | 259.00 (18.66%) | 295.00 (6.44%) | 0.38 | 188.00 (16.25%) | 172.00 (14.87%) | 0.04 |
| **Hypertension, n (%)** | 457.00 (32.93%) | 2,623.00 (57.28%) | 0.50 | 408.00 (35.26%) | 403.00 (34.83%) | 0.01 |
| **Obesity, n (%)** | 140.00 (10.09%) | 664.00 (14.50%) | 0.13 | 128.00 (11.06%) | 126.00 (10.89%) | 0.01 |
| **Spo2 mean, Median (IQR)** | 96.71 (95.24 - 98.17) | 97.90 (96.85 - 98.81) | -0.59 | 96.92 (95.56-98.32) | 97.26 (95.93-98.41) | -0.09 |
| **Glu mean, Median (IQR)** | 134.1 (109.55-170.46) | 128.20 (119.86-139.61) | -0.01 | 133.00 (108.44-169.70) | 130.92 (118.63-150.25) | -0.02 |

**Note:** MHR: Mean heart rate; MBP: Mean arterial blood pressure; MRR: Mean respiratory rate; Glu: Glucose; APS III: Acute Physiology Score III; SAPS II: Simplified Acute Physiology Score II, OASIS: Oxford Acute Severity of Illness Score, and SOFA: Sequential Organ Failure Assessment. P represent overall differences between all groups.

**Supplementary Table 6. Association between Senna and Docusate Sodium and 28-day mortality among ICU patients with sepsis**

| **Outcome** | **Models** | **HR (95%CI)** | **P** |
| --- | --- | --- | --- |
| **28-day mortality** | Propensity Score Adjusted | 0.49 (0.40-0.60) | <0.001 |
|  | Propensity Score Matched | 0.60 (0.49-0.74) | <0.001 |

This table presents the odds ratios (ORs) and 95% confidence intervals (CIs) for the association between the use of Docusate Sodium (compared to Senna) and 28-day mortality, analyzed using two approaches: propensity score-adjusted and propensity score-matched models. The propensity score-adjusted model accounts for confounding variables, while the matched model ensures balance in baseline characteristics between the two treatment groups. Statistical significance was defined as P < 0.05.
